# Supplementary material for: Surface Microbiome Profiling of Dental Elevators Using Third‐Generation Sequencing: Implications for Infection Control in Dental Practice
Source: Microbiologyopen. 2025 Dec 8;14(6):e70178. doi: 10.1002/mbo3.70178 (PMC12685768; doi:10.1002/mbo3.70178)
Supplement: Supplementary file 1 — customer_backup. [file MBO3-14-e70178-s001.zip › customer_backup/customer_backup/data_assement/M07_reads_length.pdf]

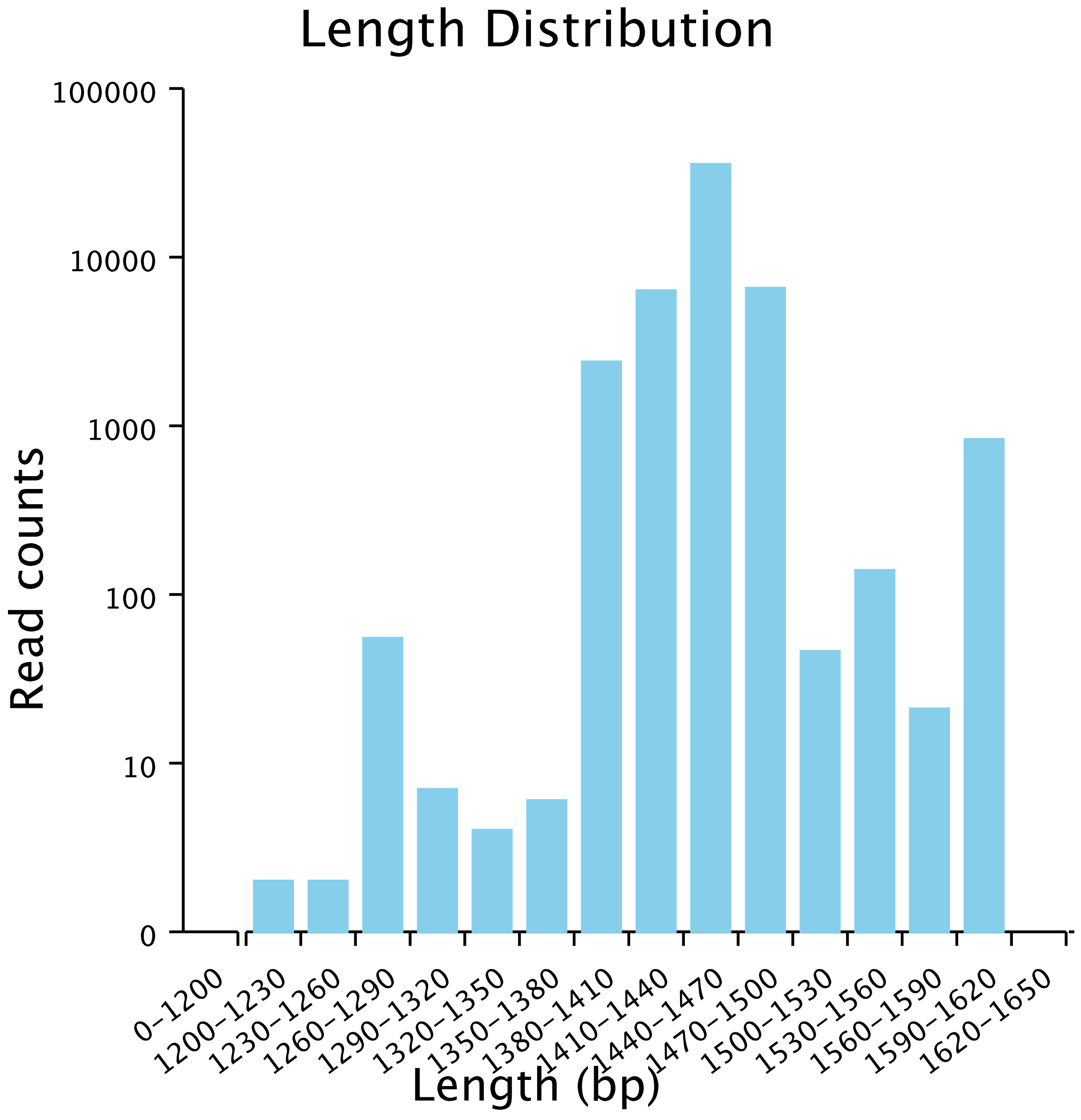

Length Distribution
Read counts
100000
10000
1000
100
10
0
0-1200
1200-1230
1230-1260
1260-1290
1290-1320
1320-1350
1350-1380
1380-1410
1410-1440
1440-1470
1470-1500
1500-1530
1530-1560
1560-1590
1590-1620
1620-1650
Length (bp)
